# Multimorbidity and adverse longitudinal outcomes among patients attending chronic outpatient medical care in Bahir Dar, Northwest Ethiopia

**DOI:** 10.3389/fmed.2023.1085888

**Published:** 2023-05-12

**Authors:** Fantu Abebe Eyowas, Marguerite Schneider, Shitaye Alemu, Fentie Ambaw Getahun

**Affiliations:** ^1^School of Public Health, College of Medicine and Health Sciences, Bahir Dar University, Bahir Dar, Ethiopia; ^2^Alan J. Flisher Centre for Public Mental Health, Department of Psychiatry and Mental Health, University of Cape Town, Cape Town, South Africa; ^3^School of Medicine, College of Medicine and Health Sciences, University of Gondar, Gondar, Ethiopia

**Keywords:** multimorbidity, quality of life, panel data, ordinal regression, Ethiopia

## Abstract

**Background:**

Multimorbidity is becoming more prevalent in low-and middle-income countries (LMICs). However, the evidence base on the burden and its longitudinal outcomes are limited. This study aimed to determine the longitudinal outcomes of patients with multimorbidity among a sample of individuals attending chronic outpatient non communicable diseases (NCDs) care in Bahir Dar, northwest Ethiopia.

**Methods:**

A facility-based longitudinal study was conducted among 1,123 participants aged 40+ attending care for single NCD (*n* = 491) or multimorbidity (*n* = 633). Data were collected both at baseline and after 1 year through standardized interviews and record reviews. Data were analyzed using Stata V.16. Descriptive statistics and longitudinal panel data analyzes were run to describe independent variables and identify factors predicting outcomes. Statistical significance was considered at *p*-value <0.05.

**Results:**

The magnitude of multimorbidity has increased from 54.8% at baseline to 56.8% at 1 year. Four percent (*n* = 44) of patients were diagnosed with one or more NCDs and those having multimorbidity at baseline were more likely than those without multimorbidity to develop new NCDs. In addition, 106 (9.4%) and 22 (2%) individuals, respectively were hospitalized and died during the follow up period. In this study, about one-third of the participants had higher quality of life (QoL), and those having higher high activation status were more likely to be in the higher versus the combined moderate and lower QoL [AOR1 = 2.35, 95%CI: (1.93, 2.87)] and in the combined higher and moderate versus lower level of QoL [AOR2 = 1.53, 95%CI: (1.25, 1.88)].

**Conclusion:**

Developing new NCDs is a frequent occurrence and the prevalence of multimorbidity is high. Living with multimorbidity was associated with poor progress, hospitalization and mortality. Patients having a higher activation level were more likely than those with low activation to have better QoL. If health systems are to meet the needs of the people with chronic conditions and multimorbidity, it is essential to understand diseases trajectories and of impact of multimorbidity on QoL, and determinants and individual capacities, and to increase their activation levels for better health improve outcomes through education and activation.

## Background

Multimorbidity is usually defined as the occurrence of two or more coexisting chronic conditions in an individual (1).

Multimorbidity is a growing global challenge with substantial impacts on individuals, health systems and the society (1). Recent reviews reported a pooled prevalence of 42.4% in high-income countries (HICs) (2), 43% in Latin America and Caribbean (3) and 36.4% in low-and middle-income countries (LMICs) (4). The magnitude of chronic multimorbidity in a recent facility based study in northwest Ethiopia was 54.8% (5).

Although mechanisms underlying the development of multimorbidity are complex, the increasing burden of multimorbidity is due to population aging and changes in lifestyle risk factors, notably physical inactivity and obesity (6). Studies in HICs have also shown that multimorbidity is socially patterned, where it mainly affects and occurred much earlier in populations with socio-economic deprivation in HICs (7, 8) and in the wealthiest quintile group in LMICs (9). The rising incidences of multimorbidity in LMICs is further influenced by the presence of adverse environmental and early life stressors linked to poverty, limited social infrastructure and poorer coping mechanisms, which ultimately lead to occurrences of chronic diseases and multimorbidity at earlier ages (1).

Non communicable diseases (NCDs) multimorbidity is associated with many adverse consequences, including death at younger age (10–12), impairments of physical and social functioning (13, 14), poor quality of life (15–17), high cost of care (18) and higher rates of adverse effects of treatment and complex interventions (19).

The management of multimorbidity is much more complicated and demanding for the health system, patients and their family (20). Although people with multiple chronic conditions require an ongoing and integrated care over a period of years or decades, they often receive a care that is fragmented and ineffective (21–24).

Despite the challenges of generating a universal management algorithm for every possible combination of chronic conditions, most models have common features (25). The overarching care principles involve integration and coordination of care, patient-centered interventions and optimization of medication therapy (25–30). Some of the models that were reported to be effective in improving outcomes of patients with multimorbidity in HICs include the patient centered medical homes (PCMH) (31), the Salford Integrated Care Program (SICP) (32), the whole system intervention (CARE Plus) (33) and patient activation (PA) (34, 35).

People who have the highest patient activation (PA) levels, including knowledge, confidence and skills to manage their own health tend to have better health outcomes than those who have a more passive approach (36, 37). Patient activation has been used to tailor self-management support interventions to improve behavioral and health-related outcomes for patients with multiple chronic conditions (38). Evidence shows higher levels of patient activation are associated with better self-management, better health outcomes, and lower healthcare costs (34). Conversely, lower patient activation scores are associated with lower QoL (38, 39). However, the authors did not find evidence on the implementation of these or other effective models of managing multimorbidity in the LMICs context. The challenges of managing multimorbidity might even be higher in LMICs where health systems are overwhelmed by high burden of communicable diseases (such as HIV, TB and Malaria) and maternal, neonatal and nutritional health problems (40). On the other hand, health systems in LIMCs are largely configured with conventional one-size fits all chronic disease care (26), which often is inadequate to meet the needs of patients with chronic multimorbidity (41). Directly applying intervention models from HICs to LMICs is not feasible as primary care is organized in different ways across countries and even within different regions of a given country (42).

In the face of struggling to fight against communicable and non-communicable diseases, and maternal childhood health problems, the emergence of multimorbidity in Ethiopia poses a serious burden to the health system. Health services in Ethiopia are largely organized around single conditions and hospital doctors who specialize in one condition or area of the body often manage patients with one condition in mind, although many people, especially as they get older, will end up with more than one diagnosed condition.

Therefore, patients with multimorbidity remain inadequately managed and suffer adverse consequences, including poor quality of life, impaired functioning, hospitalization and mortality.

Despite the huge challenge multimorbidity brings to the health system in Ethiopia, **s**ubstantial evidence gaps remain on the burden of multimorbidity, and its impacts on longitudinal patient outcomes. The need for understanding the trajectories and impacts of multimorbidity in the LMICs context has been emphasized (43, 44).

### Objective

This study aimed to determine the longitudinal outcomes of patients with multimorbidity using HRQoL as the main outcome and associated factors among a sample of individuals attending chronic outpatient NCDs care in Bahir Dar, northwest Ethiopia.

## Methods and materials

This is a multi-center facility based longitudinal study conducted both in public and private health facilities in Bahir Dar City, Ethiopia. This study is part of an ongoing research and detail of the methods applied in this study has been published elsewhere (45).

### Study setting and population

This study was conducted in five hospitals (three public and two private) and three private specialty clinics in Bahir Dar city. These facilities provide the bulk (~80%) of chronic NCDs care for the people living in the city and surrounding areas. Although chronic NCDs care and management is presumed to be provided in a relatively uniform fashion using the national NCDs treatment guideline (46), the nature of patients vising these facilities may vary and there remains a substantial difference in the quality and affordability of NCDs care between public and private health facilities in the country.

### Sample size

#### Sample size for the baseline study

The input values α (type I error = 0.05), power (1-β = 90), confidence level (95%) and the estimated non-response and attrition during follow-up (20%) were used to estimate the sample size required for measuring the variables. Compared to other methods, the sample size yielded by the general linear multivariate model with Gaussian errors (GLIMMPSE) sample size and power calculator formula (32–34) was chosen for its adequacy to answer all the quantitative study objectives. Based on the given assumptions and the approach we used, the calculated sample size required was calculated as 600. As the nature of participants is likely to be different by the type of facility (public or private) where they receive care, we employed stratification to ensure fair representation in the sample for important sub-groups analysis. Hence, a design effect of 2 was considered to avoid the possible loss of sample power during stratification. Adding 20% to the possible loss to follow-up (considering the longitudinal study) and nonresponse, the sample size needed was calculated to be 1,440.

#### Sample size for the end line study

All of the patients that were enrolled for the baseline study (*n* = 1,432, 99.4%) were approached for the end line study. However, we obtained data only from 79% (*n* = 1,123) of the participants studied at baseline. The person-time data was 2,556 as calculated through Stata to assume a longitudinal panel data.

### Sampling procedure

A two-stage stratified random sampling method was employed for recruiting facilities and participants. The sample size from each facility was determined based on the notion of probability proportional to size (PPS) using the pool of chronic NCD patients (≥ 40 yrs) registered for follow-up over the year preceding our assessment (January–December 2020) in each participating facility.

Only facilities who were providing chronic NCDs care by general practitioners or specialist physicians for at least a duration of 1 year prior to the data collection were considered. Older adults (40 years or more) diagnosed with at least one NCD and were on chronic diseases follow up care for at least 6 months prior to the study period were recruited for the study. Pregnant women and individuals who were too ill to be interviewed and admitted patients were excluded.

Participants enrolled for the baseline study (from March 15 to April 30, 2021) were invited 1 year later for the follow up study from March 15 to April 30, 2022. Contact information (mobile numbers and medical registration numbers) of patients involved in the baseline study were documented to contact them for the follow-up study. Printed copies of contact addresses of patients were given to the data collectors to sort out appointment dates of patients and to also remind patients to come for the study. All the participants agreed for the baseline study were informed about our plan to contact them 1 year from the baseline assessment. We used Kobo toolbox software to accurately match the end line data (period 2) with the baseline data (period 1) (47).

Data on QoL, patient activation (PA) score and multimorbidity were collected at two points on the same individual. However, some key outcome data such as mortality, hospitalization and perceived progress over time were collected only at the end of the follow up period.

### Definition and measurement of the primary outcome variable (HRQoL)

HRQoL (stated as QoL in this study) is defined as individuals’ perception of their position in life in the context of physical, psychological and social functioning and well-being (48). QoL at two points (baseline and end line) was measured using the interviewer-administered short form (SF-12 V2) assessment tool (49, 50).

The SF-12 tool is extensively validated and widely used generic tool for measuring QoL in multimorbidity across different contexts, including Sub-Saharan Africa (51–53). The tool was translated and pilot tested according to the study protocol we published (45). The tool measures eight health aspects, namely physical functioning (PF), role limitations due to physical health problems (RP), bodily pain (BP), general health perceptions (GH), vitality (VT), social functioning (SF), role limitations due to emotional problems (RE), and mental health (psychological distress and psychological well-being) (MH). Two summary measures are derived from the SF-12: physical health (Physical Component Summary-PCS) and mental health (Mental Component Summary-MCS). However, owing to the possibility of correlation (lack of uni-dimensionality) between the PCS and MCS scores, some studies criticized the use of these scoring algorithms and recommended raw sum scores instead (54, 55). The use of a single raw sum score enables a consistent assessment of the impact of multimorbidity and how this varies across a given population (56). Thus, we applied this approach for analyzing the QoL data.

First, we reverse coded the scores for items 1, 9 and 10 and computed the raw total. The overall scores were scaled from 0 to 100, with 0 representing worst health (57). Although popularly used in previous studies, the notion of fitting linear regression models to summarize categorical data such as the QoL data has been questioned (55, 58). The linear regression models may potentially lose important variability in the data particularly when the QoL data is collected by Liker-type scales such as the SF-12 tool (59, 60). Recent advances in the field recommend the interpretation of QoL rather as a categorical (group continuous) variable than as a metric variable (58). Studies suggest that ordinal regression models (OLR) are superior to other method for analyzing ordinal data, including health-related QoL data (58, 61). Hence, we ranked the scaled QoL scores into three ordered and non-overlapping categories as per the recommendation (60) as poor QoL (a scaled value <75), moderate QoL (scaled value from 75 to 89.9) and high QoL (scaled value from 90 to 100) and fitted into the longitudinal OLR and proportional odds (PPO) models.

### Measurement of independent variables

#### Sociodemographic characteristics

Except for age, the data on gender, education, residence and occupation were taken from the baseline records. In addition, outcomes were compared based on the baseline QoL, gender and method of data collection.

#### Non communicable diseases and NCDs multimorbidity

As explained in the study protocol (5), multimorbidity was operationalized as the co-occurrence of two or more of the chronic NCDs. List of NCDs considered in this study were determined based on our review study (62) and includes hypertension, diabetes, heart diseases (heart failure, angina and heart attack), stroke, bronchial asthma, chronic obstructive pulmonary diseases (COPD), depression and cancer.

Information on these chronic conditions was assessed through a question about ever being diagnosed with the disease by a health professional. The specific question was “have you ever been told by a health professional/doctor that you have (disease name)?” responses were either yes (scored as “1”) or no (score as “0”). Participants were also prompted to report up to three additional chronic conditions they are living with if any. To improve the quality of data obtained from interviews (63, 64), we reviewed medical records of all the study participants. At the time of the follow-up data collection, participants were asked if they are diagnosed with new (additional) NCD/s (since the baseline) and patient charts were reviewed to corroborate the information obtained from interviews if patients reported to have any. In addition to the interview and review of medical records, we used a locally validated patient health questionnaire (PHQ-9) (65) to assess mental health status. Possible PHQ-9 scores range from 0 to 27 and patients scoring 10 or more were classified as having depression (66).

In addition, data on factors potentially related to developing new NCD and multimorbidity, including age, gender and activation level were explored.

#### Patient activation score

Patient activation (PA) refers to the motivation, knowledge, skills and confidence that equip adults to be actively engaged in their health and healthcare (67). PA score was measured using validated tools (67, 68). The tool contains 13 statements answered on a 4-point Likert-type scale about managing one’s health and summed to a 100-point scale, with higher scores reflecting higher levels of activation (69). The score was classified into four stages, the lowest category being poor activation (≤47.0 = stage 1, 47.1–55.1 = stage 2, 55.2–67.0 = stage 3 and ≥ 67.1 = stage 4). We omitted the ‘not applicable’ option as it was not chosen by any of the participants at baseline.

Those who fall into Level 1 are defined as passive recipients of care who do not understand that they can play an active role in their own healthcare. Level 2 includes patients who lack the basic knowledge and confidence to effectively self-manage (for example they may not understand the treatment options available to them or what their medications do). Level 3 includes those who have a basic knowledge about their health but they lack the confidence and skills to engage in positive self-management behaviors. Level 4 is for patients who have the knowledge and confidence to self-manage but who may need support during times of personal stress or health crisis (70). The PA level has been found to be a valid and reliable measure in people with long-term conditions, including in patients with multimorbidity in different contexts (71).

### Measurement of other outcome variables

#### Hospitalization

Participants were asked if they were hospitalized (at least once) due to the chronic condition/s they are living with. Responses were recorded as yes if they were hospitalized and no if not. The factors associated with hospitalization, including the type and number of NCDs were also studied. We used binary logistic regression models adjusting for age and gender to check if NCDs and multimorbidity are associated with hospitalization.

#### Perceived progress

We asked participants to rate their progress since the baseline status. They indicated their progress (symptom burden) over time using a rating scale (poor or deteriorating progress, fair progress and very good or excellent progress) as proposed (72).

#### Mortality

We reviewed medical records and contacted patient family members (using the telephone number we recorded at baseline) to collect the mortality data.

#### Data collection tools and procedures

The tools we used to collect the baseline data were utilized to gather the follow-up data. The tools were piloted tested and standardized according to the study protocol (45). For the sake of a more efficient and accurate data collection, aggregation and statistical analysis, the follow-up data were also collected by the Kobo Toolbox software (47). Patients were interviewed and assessed following their regular consultation appointment. Physicians and nurses working in the chronic care unit were involved to facilitate the data collection process. However, data were primarily collected by graduate nurse professionals.

After obtaining consent from the participants, information on self-reported newly diagnosed medical condition/s, activation status, QoL and depression level was collected by interviewer administered questionnaires. Finally, we reviewed medical records of participants who have had a new diagnosis and those of patients reported to have died during the follow up period.

Data were collected by face-to-face interviews (*n* = 913, 81.2%) and telephone interviews (*n* = 211, 18.8%). The t-test shows no statistically significant differences in the mean age between the two method of data collection employed (*p*-value = 0.497).

#### Data quality assurance

Data were collected from multiple sources using pilot tested and standardized instruments. Eight of the 10 data collectors that were recruited for the baseline study and two newly recruited data collectors were oriented together and employed to collect the end line data. The data collection process was monitored by trained supervisors and the principal investigator. We used Kobo toolbox software to collect real time data and monitor the validity of the information uploaded to the server daily (47).

### Data analysis

The data from the Kobo toolbox server were downloaded into an excel spreadsheet and migrated to SPSS V. 21 for cleaning before being exported to Stata V. 17 for analysis. The end line data were linked to the baseline data to form the panel data.

The authors did not do imputations to account for the missing data due to the addition of a 20% sample for the possible loss during follow up and non-response and because of the probability that the missing were at random.

We ran descriptive statistics to characterize distributions of the study participants, patient reported outcomes and associated factors. All descriptive analyzes were weighted to account for the stratified sampling. In our analysis, age and social support scores were treated as continuous variables.

Sensitivity analysis was conducted using chi squared test to check whether the data collected by face-to-face interview (81.2%) and telephone interview (18.8%) have statistical difference. No difference was observed between the two based on multimorbidity status (*χ*^2^ = 7.2, *p* = 0.065) and QoL (*χ*^2^ = 2.29, *p* = 0.130) measured at baseline.

Descriptive statistics were also used to characterize and compare the distributions of PA levels and QoL levels between the baseline and end line period. For the sake of clarity, we have also computed the means and SDs of both these response variables. We checked multicollinearity of independent variables while fitting multivariable models (VIF = 1.02).

Most QoL data are measured by Likert-type scales, and the scores were treated as if they are continuous (equal distance between levels) and normally distributed (61). However, evidence shows that such data possess skewed distributions and it is unknown whether the distances between two successive or alternative levels (categories) are equal (73). Hence, analyzing ordinal data as if they are metric (continuous) can systematically lead to biased effect-size estimates, inflated errors rates and inaccurate parameter estimates (55, 74). In addition, collapsing the categories to suit for binary regression is inappropriate for ordered outcomes such as the QoL (59, 60). Hence, more sensitive and comprehensive models are required. Evidence suggests that the ordinal regression models are superior to the methods commonly used to analyze data of an ordered nature (75, 76). The ordinal models provide better theoretical interpretation and numerical inference than the metric (linear) models for ordered outcomes (77, 78). Based on this, QoL was treated as an ordered outcomes and categorized as low, moderate and high, and coded as 0, 1 and 2, respectively, as described above. However, as our data did not satisfy the parallel lines regression assumptions, we fitted the partial proportional odds model. The model treats the data in two categories (as Panel 1 vs. 2 and 3 or panel 1 and 2 vs. 3).

Our data were measured at two points in time. Considering the correlation between outcomes measure at different times, we fitted an ordered logistic panel data analysis model. Panel data analysis is useful to control unobserved characteristics that do not change over time (time invariance variables) (79).

The data measured at two periods were reshaped from wide to long format. Then, the authors set Stata to handle the longitudinal panel data by using the *xtset* command (*xtset* facility type year). We had 2,246 person time data for this analysis.

Facility type was the panel variable and year (2021 to 2022) the time variable. We obtained the following output, signifying the data were strongly balanced (all individuals have data at two times).
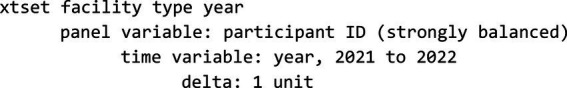


To explore the relationship between predictor and outcome variables, we fitted both fixed and random effect models.

The model we fitted is described below.

*Y_it_ = α + β_k_X_kit_ + uit + εit where*

*i = individual and t = time (from March 2021–March 2022)*

*α is the intercept*

*Y_it_ is the dependent variable (either QoL)*

*X_kit_ represents the k^th^ independent and control variable*

*Β_k_ is the coefficient for respective independent and control variables*

*uit is the impact of the i^th^ individual (not a measured variable)*

*εit is the error*

As we recruited a random sample of study participants, we performed both fixed effect and random effect regressions, then we compared them using the Hasuman test. The null hypothesis states that the error terms are not correlated. That means a significant test (*p*-value <0.05) in the Hausman test implies that the error terms are not correlated. Hence, the fixed effect model is preferred. If the test result is not significant, however, the random effect model is plausible (79). The Hausman test in our model indicates that the null hypothesis be rejected (*p*-value = 0.110). Hence, the random effect model is appropriate. We have further checked whether the random effect model is preferred to the simple OLS by running the Breusch-Pagan Lagrange Multiplier (LM) test. The null hypothesis is that variation across entities is zero (no panel effect). The LM test in our analysis showed a significant (*p*-value = 0.001) implying the random effect model is appropriate (80).

We used logistic regression analysis adjusting for age and sex to identify factors associated with development of new NCDs, multimorbidity and adverse outcomes such as hospitalization, poor progression and mortality.

## Results

### Characteristics of the study participants

Of the 1,432 participants who were enrolled in the baseline study, 1,123 (78.5%) patients agreed to participate for the follow up study. They ranged in age from 41 to 93 years (mean 57.1 ± 11.8 years, median 55 years), with a slightly higher percentage of women (50.9%) versus men (49.1%; Table 1). The primary reason for non-response were absenteeism on the date of follow-up and difficulty in tracing them *via* telephone calls (*n* = 19.06%), death of the participant (*n* = 22, 1.54%) and refusal (*n* = 9, 0.98%). There was no statistically significant difference between the attendees and the lost to follow up groups with regard to age (*p*-value = 0.504), gender (*p*-value = 0.400), multimorbidity status (*p*-value = 0.097) and the mean baseline QoL scores (*p*-value = 1.000).

**Table 1:** Socio-demographic characteristics of study participants attending chronic outpatient NCDs care in Bahir Dar, Ethiopia.

Variables	Frequency	Percentage
Age in years		
≤44	190	16.9
45–54	310	27.6
55–64	314	28.0
65+	309	27.5
Sex		
Male	552	49.1
Female	572	50.9
Residence		
Urban (Bahir Dar)	651	58.0
Other towns	287	25.5
Rural	185	16.5
Religion		
Orthodox Christian	1,033	91.9
Muslim	85	7.6
Others	6	0.5
Marital status		
Currently married	844	75.1
Single*	280	24.9
Education no		
Formal education	594	52.8
Primary	121	10.8
Secondary	145	12.9
College level and above	264	23.5
Occupation		
Housewife	267	23.8
Employed	223	19.8
Farmer	153	13.6
Trader	263	23.4
Retired	110	9.8
Unemployed	108	9.6
Wealth index (SES)		
Low	403	35.9
Middle	337	29.9
High	384	34.2
QoL		
Poor	426	37.9
Moderate	320	28.5
High	378	33.6

* Never married, divorced, widowed and separated.

### Newly diagnosed NCDs and the change in the patterns of multimorbidity

During the follow up period, 44 (3.9%) patients reported to have one or more newly diagnosed NCDs, with, a higher proportion (*n* = 34, 72.3%) of them having multimorbidity at baseline. There was a statistically significant difference in the probability of developing new NCDs between having multimorbidity and single morbidity at baseline (*p*-value = 0.003). In addition, there was a 2% increase in the magnitude of multimorbidity during the follow up period. In other words, the magnitude of multimorbidity has increased from 54.8% at baseline to 56.8% as measured at the follow up period. It was also observed that about 17% of those having multimorbidity had three or more NCDs, a 2 % increase from the baseline status (15.2%).

**Figure 1:**
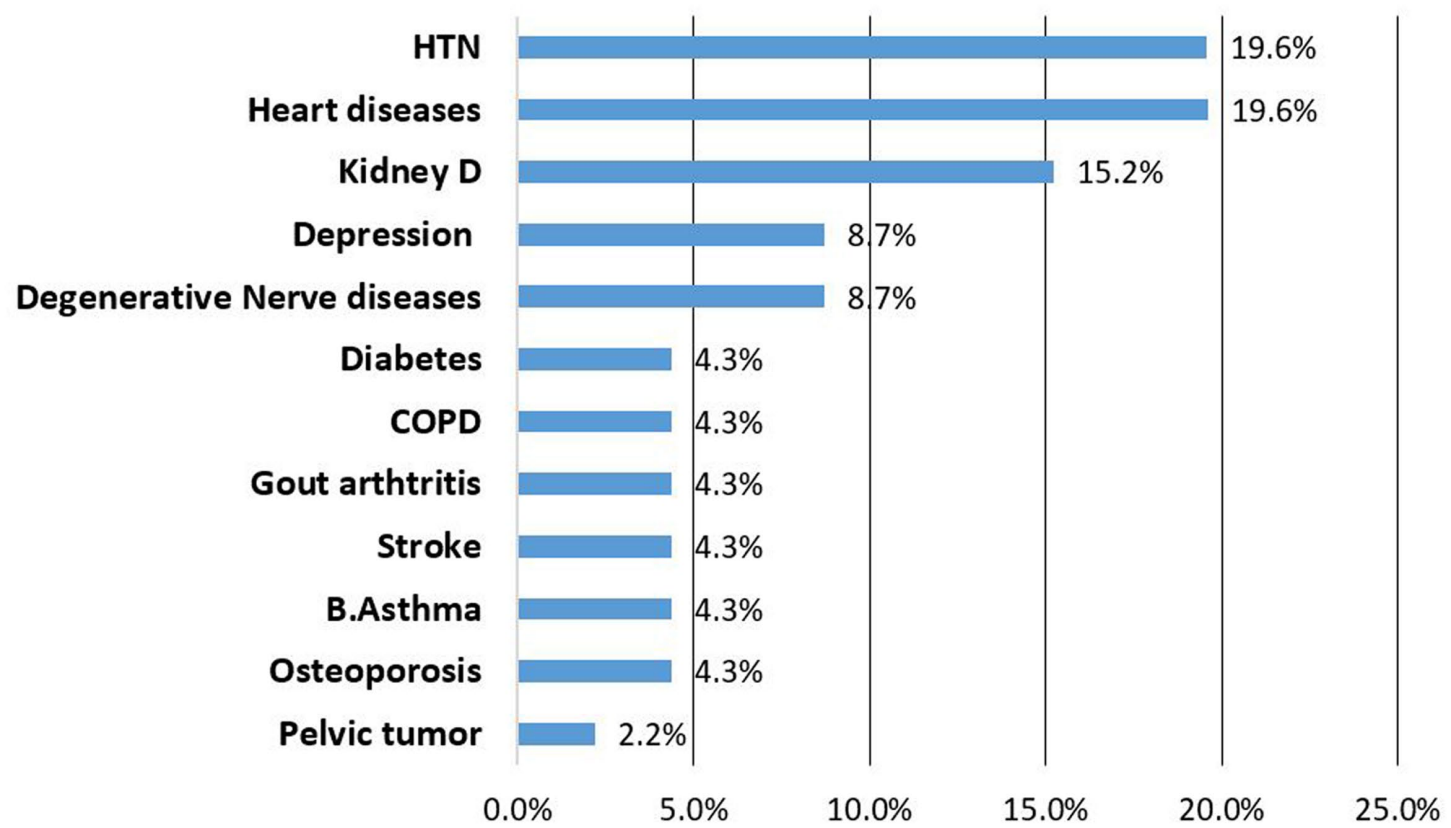
List of newly identified NCDs among patients attending NCDs care follow up, Bahir Dar Ethiopia.

Hypertension (19.6%), heat failure (19.6%) and chronic kidney diseases (15.2%) were the most frequently newly diagnosed NCDs during the follow up (Figure 1).

### Factors associated with occurrence of newly diagnosed NCDs

Having multimorbidity and being in the overweight or obese BMI category at baseline were the factors predicting development of new NCDs. However, only the presence of multimorbidity at baseline remained a statistically significant factor in the adjusted model. The odds of having a new NCD diagnosis was 2.5 times higher among patients that had multimorbidity at baseline compared to those who had single morbidity. However, participants’ age and sex were not associated with development of new NCDs during the one-year follow up (Table 2).

**Table 2:** Factors Associated with development of new NCDs among patients attending outpatient NCD care follow up, Bahir Dar Ethiopia.

Variables	New NCDs		*p*-value	Crude odds ratio (95%CI)	*p*-value	Adjusted odds ratio (95%CI)
	Yes (%)	No (%)				
Age in years			0.881	0.99 (0.97, 1.02)	-	
Sex						
Male	27 (4.9)	524 (95.1)	Base		-	
Female	17 (3)	554 (97)	0.102	0.59 (0.32, 1.11)	0.188	0.64 (0.33, 1.24)
BMI (baseline)						
Underweight	1 (0.3)	149 (99.7)	0.132	0.21 (0.03, 1.59)	0.129	0.21 (0.03, 1.60)
Normal weight	18 (3.1)	566 (96.9)	Base		-	
Overweight or obese	25 (6.6)	353 (93.4)	0.011	2.22 (1.19, 4.14)	0.153	1.61 (0.84, 3.08)
Multimorbidity at baseline						
Yes	34 (5.4)	599 (94.6)	0.004	2.81 (1.34, 5.73)	0.017*	2.52(1.18, 5.38)
No	10 (2)	481 (98)	Base		-	

*Statistically significant at *p*-value < 0.05 in the adjusted model.

### Perceived Progress

The majority (*n* = 659, 58.6%) of the participants reported that they had a good progress compared to their status at baseline. While about one third (*n* = 374, 33.3%) reported to have a fair progress and 91 patients (8.1%) had poor and deteriorating progresses.

### Hospitalization

Nearly 10% of the patients were admitted during the follow-up because of one or more of the NCDs they were living with. Presence of three or more chronic NCDs (AOR: 3.64, 95%CI = 2.15, 6.17) compared with having single NCD, and those who reported to have a deteriorating progress (AOR: 12.42, 95%CI = 6.97, 22.14) or fair progress (AOR: 2.82, 95%CI = 1.72, 4.60) since the baseline were more likely than their counterparts to have hospital admission (Table 3).

**Table 3:** Factors associated with hospitalization among patients on chronic NCDS care follow up, Bahir Dar Ethiopia.

Variables	Hospitalization		*p*-value	COR (95%)	*p*-value	AOR (95%)
	Yes	No				
Age			0.941	1.00 (0.98. 1.02)		
Morbidity						
Single NCD	33	452	Base			
2 NCDs	33	415	0.738	1.09 (0.66, 1.79)	0.811	1.06 (0.63, 1.79)
3 or more NCDs	40	151	<0.001	3.63 (2.21, 5.96)	<0.001**	3.64 (2.15, 6.17)
Perceived progress						
Poor/deteriorating	33	58	<0.001	12.36 (7.01, 21,78)	<0.001**	12.42 (6.97, 22.14)
Fair progress	44	330	<0.001	2.89 (1.78,4.72)	<0.001**	2.82 (1.72, 4.61)
Good progress	29	630	Base			
Baseline PAM level						
Level 1	16	147	0.358	1.32 (0.73, 2.41)		
Level 2	15	120	0.350	0.75 (0.40, 1.34)		
Level 3	10	95	0.796	0.89 (0.38, 2.98)		
Level 4	65	654	Base			

** Statistically significant at *p*-value < 0.001 in the adjusted model.

### Mortality

Of the total number of patients whose status was known (*n* = 1,160, 82%), 22 (2%) patients were reported to have died during the course of the one-year follow up.

### Patient activation status

The PA scores were summed and scaled into a 100-point scale to compute the mean PA value. The mean score was 76.0 ± 23.2. According to the standard classification (81), the majority (45.8%) were classified under the highest activation category (score ≥ 67.1), followed by those in level two 26.1% (score 47.1–55.1), 18.4% in level one (score < 47.0) and 9.7% in level three (score 55.2–67.0; Table 4). For the sake of simplifying the interpretation of its effect on QoL on the ordered logistic regression (proportional odds) model, we classified the PA score into two groups: high (≥55.2) and low (≤55.1). In this sense, the proportion of participants in the high and low category was 55.5 and 44.5%, respectively. We observed that activation level fluctuated over time. Based on the Pearson correlation test, the correlation between the baseline and end line PA score was 0.065, implying no strong correlation between the two measurements.

**Table 4:** Factors associated with QoL in longitudinal panel ordered logistic regression model.

Independent variables	QoL (Panels)			
	Panel one (1 Vs. 2 and 3)		Panel two (1 and 2 *Vs* 3)	
	Coefficients	AOR 1 (95%CI)	AOR2(95%CI)	*p*-value
Sex [Female vs. male (Ref)]	Constant (OR1 = OR2)	0.99 (0.82, 1.22)	0.99 (0.82, 1.22)	0.897
Age in years	Constant (OR1 = OR2)	1.00 (0.99, 1.01)	1.00 (0.99, 1.01)	0.949
Multimorbidity [yes vs.no (Ref)]	Constant (OR1 = OR2)	0.89 (0.73, 1.09)	0.89 (0.73, 1.09)	0.266
PAM [high vs. low (Ref)]	Coefficients not constant (OR1 ≠ OR2)	2.35 (1.93, 2.87)	1.53 (1.25, 1.88)	<0.001**

** Statistically significant at *p*-value < 0.05 in both panels using the random effect model.

### Quality of life

The majority (37.9%) of participants had a lower QoL and there was a modest increase from the baseline level (33.5%). In this study, about one-third (33.6) of the patients had better QoL and 28.5% of them had moderate QoL.

### Factors associated with QoL

We assessed the effect of key independent variables through fitting an ordered longitudinal panel data analysis model. As indicated in the method section above the random effect model is appropriate for the data at hand and the output of the model is shown below (Table 4).

Compared to males, females had lower odds of being in the higher level of QoL versus the combined lower and moderate QoL and in the combined higher and moderate levels of QoL versus the lower level of QoL, given the other variables are held constant in the model [AOR1 = AOR2: 0.99, (95%CI: 0.82, 1.22)]. Similarly, a person living with multimorbidity had a lower odds of being in the higher level of QoL versus the combined lower and moderate QoL and in the combined higher and moderate levels of QoL versus the lower level of QoL, given the other variables are held constant in the model [AOR1 = AOR2: 0.89, (95%CI: 0.73, 1.09)]. However, none of these association are statistically significant. Further, age has no statistically significant association with QoL in our analysis.

On the other hand, PA score has shown a statistically significant association with QoL in both panels. For individuals having a higher levels of PA score, the odd of being in the higher category of QoL versus the combined moderate and lower QoL was 2.4 times higher [AOR1: 2.35, (95%CI: 1.93, 2.87)]. Likewise, the odds of being in the combined higher and moderate QoL versus lower QoL was 1.5 times higher for individuals having a higher level of PA score [AOR2: 1.53, (95%CI: 1.25, 1.88)] (Table 4).

## Discussion

This study broadly assessed the progress and outcomes of patients attending chronic outpatient NCD care in Bahir Dar, Ethiopia.

It was found that multimorbidity is common and those having multimorbidity at baseline were more likely than individuals with single morbidity to develop new NCDs over the course of the 12 month follow up period. This may be explained by the possibility that individuals living with multimorbidity already have enough of the risk factors to developing more NCDs (44), or because of the probability that they would find it difficult to make lifestyle modifications while burdened with existing multiple conditions, or may be due to complications arising from poor management of the underlying NCDs. Other studies have also reported a higher rate of cumulative incidence of NCDs among multimorbid individuals than their counterparts in LMICs (82, 83). The findings with regard to the challenges and burdens that patients with multimorbidity face are consistent with previous studies in LMICs (1, 82).

The magnitude of multimorbidity increased from 54.8% at baseline to 56.8% by the end of the one-year follow up. A 2 % increase in the burden of multimorbidity implies that the course of developing multimorbidity is rapid and that if not properly managed, individuals with single morbidity will eventually develop multimorbidity. While living with single NCDs is challenging by itself, the addition of one or more chronic NCDs during the course of treatment may complicate patient management and result in poor clinical outcomes, including disability, poor quality of life and mortality (84). Studies have shown that timely screening and prevention of risk factors and person-centered management of index conditions help to prevent or delay occurrences of comorbidity and multimorbidity (1, 83). Although not shown in our study, previous evidence has shown that individuals with high activation level are less likely than their counterparts to develop additional morbidities during the course of their treatment (34, 69).

Monitoring the clinical progress of individuals attending chronic care is instrumental to prevent adverse outcomes and modify interventions to improve management and outcomes (82). The authors observed that the majority had higher levels of perceived progress of their conditions from the baseline status. A significant proportion (8.1%) of the participants had poor or deteriorating progress. Unless immediate action is taken, those in the latter category will suffer poor outcomes. Perhaps, those who reported to have a fair progress may progress to a deteriorating status if not managed properly. Evidence shows that carefully implemented preventive and management strategies help avoid adverse progresses and development of secondary or tertiary conditions (83, 85).

In this study, the authors found that nearly 10% of participants were hospitalized at least once during the one-year follow up due the chronic condition/s they were living with. Consistent with previous literature (86, 87), individual having multimorbidity at baseline, including those with three or more chronic conditions were more likely than individuals with single morbidity to experience hospitalization during the course of their follow up care. In agreement with previous studies (88), a dose–response relationship between the number of chronic diseases and hospitalization was also observed in this study. Other researchers argue that most multimorbidity related hospitalizations are avoidable, and their occurrence warrants a lack of care coordination and the lack of care quality, possibly because of fragmentation when addressing the problems in individuals living with multiple conditions (86).

During the one-year follow up, 22 (~2%) patients were reported to have died. Although it was difficult to ascertain the cause death of these individuals, a higher proportion (64%) of them had multimorbidity at baseline and participants’ death was not attributable to their age. Longitudinal studies consistently reported an increasing odd of mortality among individuals with multimorbidity compared to those without multimorbidity (82, 89). Higher mortality risk in those with multimorbidity indicates the need for tailored, person-centered integrated care interventions and better access to holistic healthcare for improving the wellbeing and survival of these group (90). However, in contrast to previous studies (91), neither the number of chronic conditions nor specific disease combinations are associated with mortality in our study. This might be related to the relatively short period of follow up and small sample of the deceased individuals in our analysis.

A sufficient degree of activation is required for patients with multiple chronic diseases for adequate self-management practices (92). A higher level of PA, that allows patients to take on the role of managing their own health and healthcare (70),is associated with better outcomes such as improved QoL, compliance to medication regiments, proper self-management and reduced chance of unplanned hospitalization and mortality (69, 93). In this study, it was found that the majority (55%) had higher level of PA. However, given the high and growing burden of multimorbidity, there seem a missed opportunities to enhance activation among patients attending chronic follow up care. This may result in a more rapid progression to development of more NCDs and associated complications (93).

As observed in our study, activation level may fluctuate over time and can be affected by disease progression, background of patents and quality of healthcare (94). Hence, it is important to understand individual circumstances and changes in the progression of their condition and support activation levels and behaviors sufficient to maintain their wellbeing and improve outcomes as suggested by previous literature (38).

Health related QoL is one of the outcomes that could be predicted by individuals’ activation status (95). In this study, it was found that a higher proportion (38%) of the participants had poor QoL with 33.6% reporting good QoL. Although it is not possible to determine causation, higher levels of PA predicted higher levels of QoL in our study. This finding is consistent with previous studies (37). This association illuminates a possible entry point for developing strategies to increase patient activation levels, thereby increasing QoL and improving health outcomes (95). Moreover, Racheli Magnezi et al. (95) found that patient activation intervention was particularly effective for those with PA scores at Levels 1 and 2 (i.e., the less activated patients) and any changes in PA levels were directly associated with changes in health status, with improvement in patient activation leading to better health outcomes. Activation of patients with chronic conditions can routinely be monitored and enhanced through providing instructions and specific caretaking tasks, building their confidence and encouraging patients to take additional actions, until they are finally able to manage their own conditions (81, 95).

However, unlike their effect on the QoL at baseline, age and multimorbidity did not show a statistically significant association with QoL in the longitudinal study. This variation may be partly explained by the difference in the way the survey data were handled (i.e., we used longitudinal panel data for this study and cross-sectional data at baseline) to analyze the impact of these factors on QoL at the end line. The slight changes in the levels of QoL from the baseline might have contributed to the loss of significance of these variables. Further research is need to corroborate or refute this observation.

### Implication of research, policy and practice

The main goal of health care for the people living with chronic conditions and multimorbidity is to support them achieve a better QoL, improved wellbeing and survival (28, 96). However, a significant number of patients attending chronic care in the study area experienced a range of adverse outcomes, including development of new NCDs and multimorbidity, poor disease progression, poor QoL, hospitalization and mortality. This implies living with chronic NCDs and associated multimorbidity has profound impacts on individuals, and that the health system does not seem to be well prepared to adequately respond to individual patient needs. The provision of patient-centered care in which all healthcare providers work together with patients to ensure coordination, consistency and continuity of care over time is essential (97). This will in turn improve the wellbeing and survival of the people with multimorbidity in the study area.

Given the positive association between PA level and QoL, it is desirable to determine and devise strategies to increase activation status. The notion of patient activation is relatively new in the study context and there is a need to experiment its effects in improving outcomes of patients in the chronic care landscape and beyond.

### Strength and limitations of the study

Our study has the advantage of involving a broad group of health facilities and patients receiving chronic NCDs care. Guided by a published study protocol, this longitudinal study provided strong insight on the course and patterns of disease progression, the impact of multimorbidity on important patient outcomes such as hospitalization and mortality, and the level and predictors of QoL using robust methodologies. Our ability to determine activation levels of patients would encourage service providers to measure and intervene with mechanisms to increase PA. However, the findings of this facility-based study may not exactly represent the underlying epidemiology of multimorbidity and its impact in the general population. In addition, although the sensitivity analysis does not show variations, the data collected from a portion of patients by telephone interview might not be an exact replica of the data obtained from face-to-face interviews. However, adequacy of the sample size and parsimony of the methods we employed would make our study robust.

## Conclusion and recommendations

The likelihood of developing new NCDs and multimorbidity is high. Multimorbidity is not only high in the study area, but also it associated with worst patient outcomes, including hospitalization and mortality, compared to those with single NCDs. This study revealed that the highest proportion of individuals with multimorbidity had poor QoL. On the other hand, patients having a higher level of PA level were more likely to have better levels of QoL. If health systems in LMICs are to meet the needs of the people with chronic conditions and multimorbidity, it is essential to understand the long- term, life course determinants of different multimorbidity trajectories, and to help improve individual capacity and activation levels. Replicating the evidence on the effect of patient activation on QoL and determining outcomes and predictors of people living with chronic NCDs and multimorbidity longitudinally is recommended. It is also imperative to replicate the methods that were employed to measure and analyze QoL data in this study in order to facilitate comparison and further development of the approaches.

## Data availability statement

The original contributions presented in the study are included in the article/Supplementary material, further inquiries can be directed to the corresponding author.

## Ethics statement

The studies involving human participants were reviewed and approved by College of medicine and health sciences, Bahir Dar University internal review board. Written informed consent for participation was not required for this study in accordance with the national legislation and the institutional requirements. All study subjects provided verbal consent to participate in this study.

## Author contributions

FE, FG, MS, and SA conceived and designed this study. FE, FG, and MS participated in the data analysis and interpretation of the findings. FE drafted the manuscript. FG and MS contributed in revising the manuscript. MS undertook an English language check as she is a native English speaker. All authors critically reviewed and approved the final manuscript for submission.

## Funding

This work was partially funded by Bahir Dar University with a grant number: RCS/003/20.

## Conflict of interest

The authors declare that the research was conducted in the absence of any commercial or financial relationships that could be construed as a potential conflict of interest.

## Publisher’s note

All claims expressed in this article are solely those of the authors and do not necessarily represent those of their affiliated organizations, or those of the publisher, the editors and the reviewers. Any product that may be evaluated in this article, or claim that may be made by its manufacturer, is not guaranteed or endorsed by the publisher.

## References

[1] Skou ST Mair FS Fortin M Guthrie B Nunes BP Miranda JJ. Multimorbidity. Nat Rev Dis Primers. (2022) 8:48. doi: 10.1038/s41572-022-00376-4 35835758 PMC7613517

[2] Iris Szu-Szu Ho AA-L Akbari A Davies J Hodgins P Khunti K Kadam U. Variation in the estimated prevalence of multimorbidity: systematic review and meta-analysis of 193 international studies. BMJ Open. (2022):e057017:12. doi: 10.1136/bmjopen-2021-057017 PMC9058768 35487738

[3] Huaquía-Díaz AM Chalán-Dávila TS Carrillo-Larco RM Bernabe-Ortiz A. Multimorbidity in Latin America and the Caribbean: a systematic review and meta- analysis. BMJ Open. (2021) 11:e050409. doi: 10.1136/bmjopen-2021-050409 PMC8311299 34301665

[4] Asogwa OA Boateng D Marzà-Florensa A Peters S Levitt N Jv O. Multimorbidity of non-communicable diseases in low-income and middle income countries: a systematic review and meta-analysis. BMJ Open. (2022) 12:e049133. doi: 10.1136/bmjopen-2021-049133 PMC8785179 35063955

[5] Eyowas FA Schneider M Alemu S Pati S Getahun FA. Magnitude, pattern and correlates of multimorbidity among patients attending chronic outpatient medical care in Bahir Dar, Northwest Ethiopia: the application of latent class analysis model. PLoS One. (2022) 17:e0267208. doi: 10.1371/journal.pone.0267208, PMID: 35476676 PMC9045625

[6] Freisling H Viallon V Lennon H Bagnardi V Ricci C Butterworth AS. Lifestyle factors and risk of multimorbidityof cancer and cardiometabolic diseases: amultinational cohort study. BMC Med. (2020) 18:5. doi: 10.1186/s12916-019-1474-7, PMID: 31918762 PMC6953215

[7] Mercer SW Zhou Y Humphris GM McConnachie A Bakhshi A Bikker A. Multimorbidity and socioeconomic deprivation in primary care consultations. Ann Fam Med. (2018) 16:127–31. doi: 10.1370/afm.2202, PMID: 29531103 PMC5847350

[8] Salisbury C Johnson L Purdy S Valderas JM Montgomery AA. Epidemiology and impact of multimorbidity in primary care: a retrospective cohort study. Br J Gen Pract. (2011) 61:e12–21. doi: 10.3399/bjgp11X548929 21401985 PMC3020068

[9] Roomaney RA van Wyk B Cois A Pillay-van Wyk V. Inequity in the distribution of non-communicable disease multimorbidity in adults in South Africa: an analysis of prevalence and patterns. Int J Public Health. (2022) 67. doi: 10.3389/ijph.2022.1605072, PMID: 36051505 PMC9426027

[10] Olaya B Domenech-Abella J Moneta MV Lara E Caballero FF Rico-Uribe LA. All-cause mortality and multimorbidity in older adults: the role of social support and loneliness. Exp Gerontol. (2017) 99:120–6. doi: 10.1016/j.exger.2017.10.001, PMID: 28982608

[11] Wei MY Mukamal KJ. Multimorbidity, mortality, and long-term physical functioning in 3 prospective cohorts of community-dwelling adults. Am J Epidemiol. (2018) 187:103–12. doi: 10.1093/aje/kwx198, PMID: 29309518 PMC5860284

[12] Martin-Lesende I Recalde E Viviane-Wunderling P Pinar T Borghesi F Aguirre T. Mortality in a cohort of complex patients with chronic illnesses and multimorbidity: a descriptive longitudinal study. BMC Palliat Care. (2016) 15:42. doi: 10.1186/s12904-016-0111-x, PMID: 27068572 PMC4828889

[13] Barnett K Mercer SW Norbury M Watt G Wyke S Guthrie B. Epidemiology of multimorbidity and implications for health care, research, and medical education: a cross-sectional study. Lancet. (2012) 380:37–43. doi: 10.1016/S0140-6736(12)60240-2, PMID: 22579043

[14] Wijlhuizen GJ Perenboom RJ Garre FG Heerkens YF van Meeteren N. Impact of multimorbidity on functioning: evaluating the ICF Core set approach in an empirical study of people with rheumatic diseases. J Rehabil Med. (2012) 44:664–8. doi: 10.2340/16501977-1002, PMID: 22729794

[15] Fortin M Lapointe L Hudon C Vanasse A Ntetu AL Maltais D. Multimorbidity and quality of life in primary care: a systematic review. Health Qual Life Outcomes. (2004) 2:51. doi: 10.1186/1477-7525-2-51, PMID: 15380021 PMC526383

[16] Hunger M Thorand B Schunk M Doring A Menn P Peters A. Multimorbidity and health-related quality of life in the older population: results from the German KORA-age study. Health Qual Life Outcomes. (2011) 9:53. doi: 10.1186/1477-7525-9-53, PMID: 21767362 PMC3152506

[17] Jitta DJ DeJongste MJ Kliphuis CM Staal MJ. Multimorbidity, the predominant predictor of quality-of-life, following successful spinal cord stimulation for angina pectoris. Neuromodulation: journal of the international neuromodulation. Society. (2011) 14:13–9. doi: 10.1111/j.1525-1403.2010.00321.x 21992156

[18] Picco L Achilla E Abdin E Chong SA Vaingankar JA McCrone P. Economic burden of multimorbidity among older adults: impact on healthcare and societal costs. BMC Health Serv Res. (2016) 16:173. doi: 10.1186/s12913-016-1421-7, PMID: 27160080 PMC4862090

[19] Boyd CM McNabney MK Brandt N Correa-de-Araujuo R Daniel KM Epplin J. Guiding principles for the care of older adults with multimorbidity: an approach for clinicians: American Geriatrics Society expert panel on the Care of Older Adults with multimorbidity. J Am Geriatr Soc. (2012) 60:E1–E25. doi: 10.1111/j.1532-5415.2012.04188.x 22994865 PMC4450364

[20] Tisminetzky M Bayliss EA Magaziner JS Allore HG Anzuoni K Boyd CM. Research priorities to advance the health and health Care of Older Adults with multiple chronic conditions. J Am Geriatr Soc. (2017) 65:1549–53. doi: 10.1111/jgs.14943, PMID: 28555750 PMC5507733

[21] Boehmer KR Abu Dabrh AM Gionfriddo MR Erwin P Montori VM. Does the chronic care model meet the emerging needs of people living with multimorbidity? A systematic review and thematic synthesis. PLoS One. (2018) 13:e0190852. doi: 10.1371/journal.pone.0190852, PMID: 29420543 PMC5805171

[22] Liska J Beal A. One patient is not one condition: delivering patient-centered care to those with multiple chronic conditions. Ther Innov Regul Sci. (2017) 51:468–70. doi: 10.1177/2168479017705158, PMID: 30227056

[23] McCarthy C Clyne B Corrigan D Boland F Wallace E Moriarty F. Supporting prescribing in older people with multimorbidity and significant polypharmacy in primary care (SPPiRE): a cluster randomised controlled trial protocol and pilot. Implement. Sci. (2017) 12:99. doi: 10.1186/s13012-017-0629-1, PMID: 28764753 PMC5539883

[24] Salisbury C Man MS Bower P Guthrie B Chaplin K Gaunt DM. Management of multimorbidity using a patient-centred care model: a pragmatic cluster-randomised trial of the 3D approach. Lancet. (2018) 392:41–50. doi: 10.1016/S0140-6736(18)31308-4, PMID: 29961638 PMC6041724

[25] NICE. Multimorbidity: Clinical assessment and management: Multimorbidity: Assessment, prioritisation and management of care for people with commonly occurring multimorbidity. NICE Guideline NG56. United Kingdom: National Institute for Health and Care Excellence (2016). 27683922

[26] Mercer S Salisbury C Fortin M. ABC of multimorbidity first. United Kingdom: John Wiley & Sons, Ltd. (2014).

[27] Gauvin F-P Wilson MG Lavis JN Abelson J. Citizen brief: Improving care and support for people with multiple chronic health conditions in Ontario. Hamilton, Canada: McMaster Health Forum (2014).

[28] Aiden H. Multimorbidity. A report for the Richmond Group of Charities: Understanding the challenge (2018).

[29] Salisbury C. Multimorbidity: redesigning health care for people who use it. Lancet. (2012) 380:7–9. doi: 10.1016/S0140-6736(12)60482-6, PMID: 22579042

[30] Stokes J Man MS Guthrie B Mercer SW Salisbury C Bower P. The foundations framework for developing and reporting new models of Care for Multimorbidity. Ann Fam Med. (2017) 15:570–7. doi: 10.1370/afm.2150, PMID: 29133498 PMC5683871

[31] Swietek KE Domino ME Beadles C Ellis AR Farley JF Grove LR. Do medical homes improve quality of Care for Persons with multiple chronic conditions? Health Serv Res. (2018) 53:4667–81. doi: 10.1111/1475-6773.13024, PMID: 30088272 PMC6232445

[32] Bower P Reeves D Sutton M Lovell K Blakemore A Hann M. Improving care for older people with long-term conditions and social care needs in Salford: the CLASSIC mixed-methods study, including RCT. Health Serv. Deliv. Res. (2018) 6:1–188. doi: 10.3310/hsdr06310, PMID: 30183219

[33] Mercer SW Fitzpatrick B Guthrie B Fenwick E Grieve E Lawson K. The CARE plus study - a whole-system intervention to improve quality of life of primary care patients with multimorbidity in areas of high socioeconomic deprivation: exploratory cluster randomised controlled trial and cost-utility analysis. BMC Med. (2016) 14:88. doi: 10.1186/s12916-016-0634-2, PMID: 27328975 PMC4916534

[34] Blakemore A Hann M Howells K Panagioti M Sidaway M Reeves D. Patient activation in older people with long-term conditions and multimorbidity: correlates and change in a cohort study in the United Kingdom. BMC Health Serv Res. (2016) 16:582. doi: 10.1186/s12913-016-1843-2, PMID: 27756341 PMC5069882

[35] Hibbard JH Greene J Shi Y Mittler J Scanlon D. Taking the long view: how well do patient activation scores predict outcomes four years later? Med Care Res Rev. (2015) 72:324–37. doi: 10.1177/1077558715573871 25716663

[36] Richards SH Dickens C Anderson R Richards DA Taylor RS Ukoumunne OC. Assessing the effectiveness of enhanced psychological care for patients with depressive symptoms attending cardiac rehabilitation compared with treatment as usual (CADENCE): a pilot cluster randomised controlled trial. Trials. (2018) 19:211. doi: 10.1186/s13063-018-2576-9, PMID: 29609644 PMC5880097

[37] Magadi W Lightfoot CJ Memory KE Santhakumaran S van der Veer SN Thomas N. Patient activation and its association with symptom burden and quality of life across the spectrum of chronic kidney disease stages in England. BMC Nephrol. (2022) 23:45. doi: 10.1186/s12882-022-02679-w, PMID: 35081904 PMC8793272

[38] Newland P Lorenz R Oliver BJ. Patient activation in adults with chronic conditions: a systematic review. J Health Psychol. (2021) 26:103–14. doi: 10.1177/1359105320947790 32830587

[39] Remmers C Hibbard J Mosen DM Wagenfield M Hoye RE Jones C. Is patient activation associated with future health outcomes and healthcare utilization among patients with diabetes? J Ambulatory Care Manage. (2009) 32:320–7. doi: 10.1097/JAC.0b013e3181ba6e77 19888008

[40] WHO. Multimorbidity: Technical Series on Safer Primary Care. Geneva: WHO (2016).

[41] Heide I Snoeijs S Melchiorre MG Quattrini S Boerma W Schellevis F. Innovating care for people with multiple chronic conditions in Europe. (2015).

[42] Eyowas FA Schneider M Yirdaw BA Getahun FA. Multimorbidity of chronic noncommunicable diseases and its models of care in low- and middle-income countries: a scoping review protocol. BMJ Open. (2019) 9:e033320. doi: 10.1136/bmjopen-2019-033320 PMC6797258 31619434

[43] AMS. Advancing research to tackle multimorbidity: the UK and LMIC perspectives. Premstätten: AMS (2018).

[44] Cezard G McHale CT Sullivan F Bowles JKF Keenan K. Studying trajectories of multimorbidity: a systematic scoping review of longitudinal approaches and evidence. BMJ Open. (2021) 11:e048485. doi: 10.1136/bmjopen-2020-048485, PMID: 34810182 PMC8609933

[45] Eyowas FA Schneider M Alemu S Getahun FA. Multimorbidity of chronic noncommunicable diseases: burden, care provision and outcomes over time among patients attending chronic outpatient medical care in Bahir Dar, Ethiopia—a mixed methods study protocol. BMJ-Open. (2021) 11:e051107. doi: 10.1136/bmjopen-2021-051107, PMID: 34497085 PMC8438962

[46] Michael M Dagnaw W Yadeta D Feleke Y Fantaye A Kebede T. *Ethiopian National Guideline on Major NCDs*. (2016).

[47] OCHA. *Manual Kobo Toolbox. Office for the Coordination of Humanitarian Affairs (OCHA) in West and Central Africa*. (2019). Available at: https://www.kobotoolbox.org/.

[48] Skevington SM Lotfy M O’Connell KA. The World Health Organization’s WHOQOL-BREF quality of life assessment: psychometric properties and results of the international field trial A report from the WHOQOL group. Qual Life Res. (2004) 13:299–310. doi: 10.1023/B:QURE.0000018486.91360.00, PMID: 15085902

[49] Gonzalez-Chica DA Hill CL Gill TK Hay P Haag D Stocks N. Individual diseases or clustering of health conditions? Association between multiple chronic diseases and health-related quality of life in adults. Health Qual Life Outcomes. (2017) 15:244. doi: 10.1186/s12955-017-0806-6, PMID: 29268792 PMC5740772

[50] Carlozzi NE Kratz AL Downing NR Goodnight S Miner J Migliore N. Validity of the 12-item World Health Organization disability assessment schedule 2.0 (WHODAS 2.0) in individuals with Huntington disease (HD). Qual Life Res. (2015) 24:1963–71. doi: 10.1007/s11136-015-0930-x, PMID: 25636661 PMC4497948

[51] Williams JS Egede LE. The association between multimorbidity and quality of life, health status and functional disability. Am J Med Sci. (2016) 352:45–52. doi: 10.1016/j.amjms.2016.03.004 27432034

[52] Ware JEJ Kosinski MM Keller SD. A 12-item short-form health survey: construction of scales and preliminary tests of reliability and validity. Med Care. (1996) 34:220–33. doi: 10.1097/00005650-199603000-00003 8628042

[53] Ohrnberger J Anselmi L Fichera E Sutton M. Validation of the SF12 mental and physical health measure for the population from a low-income country in sub-Saharan Africa. Health Qual Life Outcomes. (2020) 18:78. doi: 10.1186/s12955-020-01323-1, PMID: 32188461 PMC7081543

[54] Hagell P Westergren A Årestedt K. Beware of the origin of numbers: standard scoring of the SF-12 and SF-36 summary measures distorts measurement and score interpretations. Res Nurs Health. (2017) 40:378–86. doi: 10.1002/nur.21806, PMID: 28732149

[55] McKenna SP Heaney A. Composite outcome measurement in clinical research: the triumph of illusion over reality? J Med Econ. (2020) 23:1196–204. doi: 10.1080/13696998.2020.1797755, PMID: 32673124

[56] Lawson KD Mercer SW Wyke S Grieve E Guthrie B Watt GC. Double trouble: the impact of multimorbidity and deprivation on preference-weighted health related quality of life a cross sectional analysis of the Scottish health survey. Int J Equity Health. (2013) 12:67. doi: 10.1186/1475-9276-12-67, PMID: 23962150 PMC3765174

[57] Stubbs B Vancampfort D Veronese N Kahl KG Mitchell AJ Lin PY. Depression and physical health multimorbidity: primary data and country-wide meta-analysis of population data from 190 593 people across 43 low- and middle-income countries. Psychol Med. (2017) 47:2107–17. doi: 10.1017/S0033291717000551, PMID: 28374652

[58] Lall R. *The application of ordinal regression models in quality of life scales used in gerontology*. (2004).

[59] Lall R Campbell MJ Walters SJ Morgan K. A review of ordinal regression models applied on health-related quality of life assessments. Stat. Methods Med. Res. (2002) 11:49–67. doi: 10.1191/0962280202sm271ra 11923993

[60] Abreu MNS Siqueira AL Cardoso CS Caiaffa WT. Ordinal logistic regression models: application in quality of life studies. Cad Saúde Pública. (2008) 24:S581–91. doi: 10.1590/s0102-311x2008001600010 18797732

[61] Walters SJ Campbell MJ Lall R. Design and analysis of trials with quality of life as an outcome: a practical guide. J Biopharm Stat. (2001) 11:155–76. doi: 10.1081/BIP-100107655 11725929

[62] Abebe F Schneider M Asrat B Ambaw F. Multimorbidity of chronic non-communicable diseases in low- and middle-income countries: A scoping review. J Comorb. (2020) 10:2235042X2096191–13. doi: 10.1177/2235042X20961919 PMC7573723 33117722

[63] Fortin M Haggerty J Sanche S Almirall J. Self-reported versus health administrative data: implications for assessing chronic illness burden in populations. A cross-sectional study. CMAJ Open. (2017) 5:E729–33. doi: 10.9778/cmajo.20170029, PMID: 28947426 PMC5621946

[64] Byles JE D'Este C Parkinson L O'Connell R Treloar C. Single index of multimorbidity did not predict multiple outcomes. J Clin Epidemiol. (2005) 58:997–1005. doi: 10.1016/j.jclinepi.2005.02.025 16168345

[65] Gelaye B Williams MA Lemma S Deyessa N Bahretibeb Y Shibre T. Validity of the patient health Questionnaire-9 for depression screening and diagnosis in East Africa. Psychiatry Res. (2013) 210:653–61. doi: 10.1016/j.psychres.2013.07.015 23972787 PMC3818385

[66] Ambaw F Mayston R Hanlon C Alem A. Depression among patients with tuberculosis: determinants, course and impact on pathways to care and treatment outcomes in a primary care setting in southern Ethiopia—a study protocol. BMJ Open. (2015) 5:e007653. doi: 10.1136/bmjopen-2015-007653, PMID: 26155818 PMC4499723

[67] Hibbard JH Stockard J Mahoney ER Tusler M. Development of the patient activation measure (PAM): conceptualizing and measuring activation in patients and consumers. Health Serv Res. (2004) 39:1005–26. doi: 10.1111/j.1475-6773.2004.00269.x, PMID: 15230939 PMC1361049

[68] Schmaderer M Pozehl B Hertzog M Zimmerman L. Psychometric properties of the patient activation measure in multimorbid hospitalized patients. J Nurs Meas. (2015) 23:128–41. doi: 10.1891/1061-3749.23.3.E128, PMID: 26673761

[69] Mosen DM Schmittdiel J Hibbard J Sobel D Remmers C Bellows J. Is patient activation associated with outcomes of Care for Adults with Chronic Conditions? J Ambul Care Manage. (2007) 30:21–9. doi: 10.1097/00004479-200701000-00005 17170635

[70] Hibbard JH Mahoney ER Stockard J Tusler M. Development and testing of a short form of the patient activation measure. Health Serv Res. (2005) 40:1918–30. doi: 10.1111/j.1475-6773.2005.00438.x, PMID: 16336556 PMC1361231

[71] Skolasky RL Green AF Scharfstein D Boult C Reider L Wegener ST. Psychometric properties of the patient activation measure among multimorbid older adults. Health Serv Res. (2011) 46:457–78. doi: 10.1111/j.1475-6773.2010.01210.x, PMID: 21091470 PMC3064914

[72] Madans JH Webster KM. Health status: International encyclopedia of the Social and Behavioral Sciences. 2nd ed (2015).

[73] Preisser JS Phillips C Perin J Schwartz TA. Partial proportional odds models for longitudinal ordinal data. Amsterdam, Netherlands: Elsevier (2010).

[74] Liddell TM Kruschke JK. Analyzing ordinal data with metric models: what could possibly go wrong? J Exp Soc Psychol. (2018) 79:328–48. doi: 10.1016/j.jesp.2018.08.009

[75] Williams R. Understanding and interpreting generalized ordered logit models. J Math Sociol. (2016) 40:7–20. doi: 10.1080/0022250X.2015.1112384

[76] Peterson B Frank E Harrell J. Partial proportional odds models for ordinal response variables. J R Stat Soc. (1990) 39:205–17. doi: 10.2307/2347760

[77] Williams R. Generalized ordered logit/partial proportional odds models for ordinal dependent variables. Stata J. (2006) 6:58–82. doi: 10.1177/1536867X0600600104

[78] Bürkner PC Vuorre M. Ordinal regression models in psychology: a tutorial. Adv Methods Pract Psychol Sci. (2019) 2019:1–25. doi: 10.1177/2515245918823199

[79] Torres-Reyna O. *Panel data analysis fixed and random effects using Stata*. (2007).

[80] Xu D Lee SH Xu H. Introduction to panel data analysis. Cambridge, MA: Academic Publishers (2007).

[81] Hibbard JH. Patient activation and the use of information to support informed health decisions. Patient Educ Couns. (2017) 100:5–7. doi: 10.1016/j.pec.2016.07.006 27432014

[82] Odland ML Ismail S Sepanlou SG Poustchi H Sadjadi A Pourshams A. Multimorbidity and associations with clinical outcomes in a middle- aged population in Iran: a longitudinal cohort study. BMJ Glob Health. (2022) 7:e007278. doi: 10.1136/bmjgh-2021-007278, PMID: 35550337 PMC9109019

[83] Shang X Peng W Hill E Szoeke C Heand M Zhang L. Incidence, progression, and patterns of multimorbidity in community dwelling middle-aged men and women. Front Public Health. (2020) 8:404. doi: 10.3389/fpubh.2020.00404 33014956 PMC7461897

[84] Garin N Olaya B Moneta MV Miret M Lobo A Ayuso-Mateos JL. Impact of multimorbidity on disability and quality of life in the Spanish older population. PLoS One. (2014) 9:e111498. doi: 10.1371/journal.pone.0111498, PMID: 25375890 PMC4222819

[85] Rijken M Hujala A Ev G Melchiorre MG Groenewegen P Schellevis F. Managing multimorbidity: profiles of integrated care approaches targeting people with multiple chronic conditions in Europe夽. Health Policy. (2018) 122:44–52. doi: 10.1016/j.healthpol.2017.10.002, PMID: 29102089

[86] Nunes BP Soares MU Wachs LS Volz PM Saes MO Duro SMS. Hospitalization in older adults: association with multimorbidity, primary health care and private health plan. Rev Saude Publica. (2017) 51:43. doi: 10.1590/s1518-8787.2017051006646 28492761 PMC5433790

[87] Wang XX Chen ZB Chen XJ Huang LL Song XY Wu X. Functional status and annual hospitalization in multimorbid and non-multimorbid older adults: a cross-sectional study in southern China. Health Qual Life Outcomes. (2018) 16:33. doi: 10.1186/s12955-018-0864-4, PMID: 29433527 PMC5809886

[88] Chung RY Mercer SW Yip BH Chan SW Lai FT Wang HH. The association between types of regular primary care and hospitalization among people with and without multimorbidity: A household survey on 25,780 Chinese. Sci Rep. (2016) 6:29758. doi: 10.1038/srep29758, PMID: 27435519 PMC4951721

[89] Wade AN Payne CF Berkman L Chang A Olivé XG Kabudula C. Multimorbidity and mortality in an older, rural black south African population cohort with high prevalence of HIV findings from the HAALSI study. BMJ Open. (2021) 11:e047777. doi: 10.1136/bmjopen-2020-047777, PMID: 34526338 PMC8444254

[90] Kabir A Ansari S Conway DP Barr M. Impact of multimorbidity and complex multimorbidity on mortality among older Australians aged 45 years and over: a large population- based record linkage study. BMJ Open. (2022) 12:e060001. doi: 10.1136/bmjopen-2021-060001 PMC9330333 35882467

[91] He K Zhang W Hu X Zhao H Guo B Shi Z. Relationship between multimorbidity, disease cluster and all-cause mortality among older adults: a retrospective cohort analysis. BMC Public Health. (2021) 21:1080. doi: 10.1186/s12889-021-11108-w 34090390 PMC8180153

[92] Zimbudzi E Lo C Ranasinha S Fulcher GR Jan S Kerr PG. Factors associated with patient activation in an Australian population with comorbid diabetes and chronic kidney disease: a cross-sectional study. BMJ Open. (2017) 7:e017695. doi: 10.1136/bmjopen-2017-017695, PMID: 29061622 PMC5665291

[93] Kenning C Coventry PA Gibbons C Bee P Fisher L Bower P. Does patient experience of multimorbidity predict self-management and health outcomes in a prospective study in primary care? Fam Pract. (2015) 32:311–6. doi: 10.1093/fampra/cmv002, PMID: 25715962 PMC4445135

[94] Schmaderer MS Zimmerman L Hertzog M Pozehl B Paulman A. Correlates of patient activation and acute care utilization among multimorbid patients. West J Nurs Res. (2016) 38:1335–53. doi: 10.1177/0193945916651264, PMID: 27245080

[95] Magnezi R Glasser S Shalev H Sheiber A Reuveni H. Patient activation, depression and quality of life. Patient Educ Couns. (2014) 94:432–7. doi: 10.1016/j.pec.2013.10.015 24331277

[96] Smith SM Wallace E Salisbury C Sasseville M Bayliss E Fortin M. A Core outcome set for multimorbidity research (COSmm). Ann Fam Med. (2018) 16:132–8. doi: 10.1370/afm.2178, PMID: 29531104 PMC5847351

[97] Valderas JM Gangannagaripalli J Nolte E Boyd CM Roland M Sarria-Santamera A. Quality of care assessment for people with multimorbidity, scoping review (2019) 285:289–300. doi: 10.1111/joim.12881, 30719790

